# The fate of lysine: Non-targeted stable isotope analysis reveals parallel ways for lysine catabolization in *Phaeobacter inhibens*

**DOI:** 10.1371/journal.pone.0186395

**Published:** 2017-10-23

**Authors:** Lorenz C. Reimer, Sabine E. Will, Dietmar Schomburg

**Affiliations:** Department of Bioinformatics and Biochemistry, Technische Universität Braunschweig, Braunschweig, Germany; University of Münster, GERMANY

## Abstract

For a detailed investigation of the degradation of lysine in *Phaeobacter inhibens* DSM 17395, stable isotope experiments with uniformly ^13^C labeled L-lysine were carried out with lysine adapted cells and the metabolites were analyzed using GC/MS and HPLC/MS. A non-targeted stable isotope analysis was used which compares labeled and not labeled samples to determine the Mass Isotopomer Distribution not only for known metabolites but for all labeled compounds in our GC/MS analysis. We show that *P*. *inhibens* uses at least two parallel pathways for the first degradation steps of lysine. Further investigations identified L-pipecolate as an L-lysine degradation intermediate in *P*. *inhibens*. The analysis of HPLC/MS data as well as the labeling data of tricarboxylic acid (TCA) cycle intermediates show that L-lysine is not only catabolized directly to acetyl-CoA but also via the ethylmalonyl-CoA-pathway, leading to entry points into the TCA cycle via acetyl-CoA, succinyl-CoA, and malate. Altogether the presented data give a detailed insight into the catabolization of L-lysine following the fate of ^13^C labeled carbon via several ways into the TCA cycle.

## Introduction

*Phaeobacter inhibens* DSM 17395 is a member of the *Roseobacter* group, which comprises a large number of physiologically diverse members [1]. Its members are classified as *Alphaproteobacteria* and belong to the family of *Rhodobacteraceae*. Members of the *Roseobacter* group are globally distributed and are often isolated from algae, like *Dinoroseobacter shibae* from *Prorocentrum lima* [2] or *P*. *inhibens* 2.10 from *Ulva australis* [3]. This observation might indicate a symbiosis between the bacteria and the phytoplankton [4,5], which also explains the metabolic versatility of some of the *Roseobacter* group members, favoring the growth under nutrient-rich conditions like collapsing algal blooms.

The remarkable high versatility of *P*. *inhibens* DSM 17395 with regard to its metabolism as shown by the detailed analysis of the metabolism of cells grown on complex media [6] or on defined media containing carbohydrates [7] or amino acids [8], emphasizes the bacterium as an ideal model organism for marine bacteria isolated from a harbor environment [9] and therefore exposed to quickly-changing nutrient conditions. In a recent study [10], cells grown on nine different amino acids (tryptophan, phenylalanine, methionine, leucine, isoleucine, valine, histidine, lysine, and threonine) were studied by a combination of proteomics, metabolomics, and enzymatic analysis. These experiments indicated that lysine is not degraded via the common saccharopine pathway but rather via parallel pathways involving either 5-aminopentanoate or 2-aminoadipate.

Lysine degradation, in general, is one of the most diverse catabolic processes known for amino acid degradation. In the Metacyc database [11], eleven different pathways are annotated, most of them aerobic. Six of these pathways can be found in bacteria, while three pathways are found in fungi and two in mammals. Almost all of them end at glutaryl-CoA (anterior pathway) and continue with the degradation of glutaryl-CoA to acetyl-CoA (posterior pathway). While in higher animals and plants the major route used for the anterior pathway is the one via saccharopine [12], where a transamination to 2-oxoglutarate is involved, in bacteria a number of different pathways are used. *Escherichia coli* and *Pseudomonas aeruginosa* decarboxylate lysine to cadaverine. In case of *E*. *coli*, cadaverine is not further catabolized but exported to increase the extracellular pH during acid stress [13]. In *P*. *aeruginosa* cadaverine is converted to 5-aminopentanoate, which is degraded via glutarate and glutaryl-CoA to acetyl-CoA [14]. This is also the pathway described for *P*. *inhibens* [10]. Another bacterium that is able to catabolize lysine via multiple pathways is *Pseudomonas putida*. Revelles et al [15] described that one degradation pathway for *P*. *putida* also involves 5-aminopentanoate. In contrast to the cadaverine pathway in *P*. *aeruginosa*, the pathway in *P*. *putida* starts with an oxidation of L-lysine to 5-aminopentamide followed by a hydrolysis to 5-aminopentanoate. The second pathway for *P*. *putida* starts with a racemase which transforms L-lysine to D-lysine followed by a conversion via L-pipecolate to 2-aminoadipate. Drueppel et al [10] described in *P*. *inhibens* another pathway of lysine conversion to 2-aminoadipate, in which an aminogroup is transferred from L-lysine to pyruvate and 2-aminoadipate-6-semialdehyde is produced, which is then oxidized to 2-aminoadipate.

Although two potential anterior pathways are proposed for *P*. *inhibens*, it remains unclear whether *P*. *inhibens* is actively using both pathways to degrade L-lysine or if even additional pathways are used. To investigate the details of L-lysine degradation, we performed a comprehensive stable-isotope analysis using uniformly ^13^C labeled L-lysine spiked into *P*. *inhibens* cultures that were adapted to lysine as the sole carbon source.

Over the past decades, isotope analysis has proven to be the key for understanding metabolic processes in detail, as shown in the elucidation of the Calvin-Benson cycle [16] and the Entner-Doudoroff pathway [17] using radioactive ^14^C isotopes. By tracking the fate of an isotopically enriched precursor in the metabolism, this method provides the option to analyze its catabolization and entrance in the central metabolism. However, most experiments using isotope analyses were often restricted to a limited number of metabolites by the applied method and often only one or two metabolites were investigated.

Recent progress in the analytical techniques not only allows the analysis of stable isotopes like ^13^C and ^15^N but also increases the number of analyzable metabolites to a large extent. In combination with the development in the computational modeling of pathways, the analysis of stable isotopes has become the basis for the calculation of metabolic fluxes, which is not only used to elucidate but also to optimize metabolic fluxes [18]. In most cases published so far, stable isotope analysis is restricted to the proteogenic amino acids which are obtained in high yields by hydrolysis of proteins. Due to their low concentrations and high variability, intracellular metabolites are often neglected, although the analysis of the distribution of stable isotopes in all measurable metabolites provides a much more detailed picture of the metabolic fluxes in a cell.

Recently, the field of stable isotope analysis was extended to non-targeted approaches enabled by software like NTFD, mzMatch-ISO and X^13^CMS [19–21] which are able to determine the mass isotopomer distribution (MID) of all peaks, independent of their identification. The determination of labeling information for all measurable metabolites greatly enhances the possibilities of stable isotope analysis, which will not only help the understanding of processes in known pathways but also will help to find so far unknown pathways and unknown metabolites.

## Materials and methods

### Bacterial strains and media

*P*. *inhibens* DSM 17395 wild type and Δ262kb plasmid curing mutant [22] were obtained from the Deutsche Sammlung von Mikroorganismen und Zellkulturen GmbH (DSMZ, Braunschweig, Germany). For the cultivation, the previously described defined mineral medium [23] was used. 15 mM L-lysine was used as the sole carbon source. For labeling experiments uniformly ^13^C_6_ labeled L-lysine was used with a purity of 99% (Campro Scientific, Berlin, Germany).

### Cultivation

A single glycerol stock of *P*. *inhibens* DSM 17395 was used to inoculate pre-cultures of 20 mL medium in 100 mL Erlenmeyer flasks with three baffles. At approximately 1/2 maximum optical density (1/2 OD_max_), a new culture of 50 mL medium in 300 mL Erlenmeyer flasks with 3 baffles was inoculated with 1.5% v/v of the preculture. Another up-scaling step was performed at 1/2 OD_max_ by inoculation of 250 mL medium in 1 L Erlenmeyer flasks with 3 baffles with 8% v/v of the second culture. All cultures were incubated on a rotary shaker (150 rpm, Sartorius BS1) at 28°C.

Bacterial growth was monitored by measuring the optical density at 600 nm (Thermo Fisher UV6).

### Spiking

Spiking was performed in the middle of the linear growth phase (S1 Table). For spiking with U^13^C_6_ L-lysine, flasks were shortly removed from the incubator, the lysine was added and the flasks were placed back to the incubator to prevent stress by temperature changes and oxygen limitations. Growth data (S1 Table “60min labeling experiment”) show no influence of the spiking on the growth. As no suitable quenching method is available for *P*. *inhibens*, short time labeling experiments to determine kinetics in the processing of the substrate were not possible. The optimal labeling time, as well as the ratio of labeled to unlabeled lysine were determined in preliminary experiments. As a result, 15 minutes showed to be suitable to see changes in the anterior lysine degradation and 60 minutes were chosen for the analysis of the posterior lysine degradation. Regarding the increasing complexity of the interpretation of labeling patterns beyond the TCA cycle, no long-term labeling experiments were conducted.

### Harvesting and metabolite extraction

Harvesting of the cells was performed during exponential growth phase at 1/2 OD_max_. For GC/MS, three biological replicates with two technical replicates each were harvested. The culture volume (mL) necessary for the analysis containing 20 mg dry mass was calculated from the OD (49.2 / (OD_600nm_—0.06). Harvesting was performed as previously described [23] with the following modifications: centrifugation was applied over 10 minutes at 30.000 xg and 2°C. For washing steps, 3.5% (w/v) sodium chloride solution was used. For HPLC/MS samples, two biological replicates, each with two technical replicates, were analyzed. 10 mg dry mass were harvested by centrifugation for 10 minutes at 30.000 xg and 2°C. Cells were re-suspended in 1 ml methanol, which contained 0.2 mg∙l^-1^ biochanin A as internal standard. The suspensions were transferred into 2 ml Precellys tubes (Peqlab, Germany) containing 0.6 g ± 0.03 glass beads (70–110 μm diameter; Kuhmichel Abrasiv GmbH, Germany) and frozen in liquid nitrogen.

CoA-esters were isolated from frozen cell pellets. Cells were lysed using a Precellys 24 homogenizer (Peqlab, Germany) at -10°C. The procedure included three cycles of homogenization (6800 rpm, 30 s with equivalent breaks). The lysate was transferred to 10 ml of ice-cold ammonium acetate (25 mM, pH 6) and centrifuged (5 min at 10,000 g, 4°C). CoA-derivatives were extracted on a Strata XL-AW solid phase extraction column (Phenomenex, Germany). The column was equilibrated with 1 ml methanol followed by 1 ml methanol: H_2_O: formic acid (50: 45: 5) and 1 ml H_2_O prior sample application. Cell lysate was applied onto the column (800–900 mbar vacuum, 5 min), washed with 1 ml ammonium acetate (25 mM, pH 7.2) and 1 ml methanol, followed by 3 min of drying at 700 mbar vacuum. CoA-esters were eluted two times with 500 μl methanol containing 2% (v/v) ammonia solution (27% ammonia in H_2_O). The eluate fractions were dried in a vacuum concentrator (SpeedVac, Labconco, USA) with rotation (15°C overnight).

### Derivatization and GC/MS measurements

For the dried GC/MS samples, a two-step derivatization reaction was performed as described before [8]. Within this first step, oximes are formed when methoxyamine (Meox) reacts with carbonyl groups. During the second step (trimethylsilyl)-trifluoroacetamide (TMS) substitutes active hydrogens by silylation, thereby multiple derivates of one metabolite can be formed. Measurements were done on a Thermo GC Ultra coupled to a DSQ II mass spectrometer and on a Leco Pegasus 4D GCxGC TOFMS (in one-dimensional mode) combined with an Agilent 7890A as described before [24,25]. In addition to the split mode described by Abu Dawud [24] for measurements with the DSQ II, samples were injected in splitless mode and the MS was tuned for high masses.

### HPLC measurements of coenzyme A-derivatives

Buffers, eluents, column and flow rates for HPLC-MS analysis of CoA-derivatives were used as described by Peyraud et al. [26]. Dried extracts were resolved in 200 μl sample buffer (25 mM ammonium acetate pH 3.5, 2% methanol) and 50 μl were injected on a Dionex ultimate 3000 system (Thermo Scientific Inc., Germany) coupled to a Bruker MicroTOF QII mass spectrometer (Bruker Daltonik GmbH, Germany) equipped with an electrospray ionisation interface. Measurements were carried out as described by Wolf et al. [27].

### HPLC data processing and peak identification

Data export to mzXML format and internal mass calibration using the sodium formate cluster was carried out with DataAnalysis software (version 4.0 SP 5 (Build 283), Bruker Daltonik GmbH, Germany). Raw data were processed using the XCMS package [28–30] for R (version 3.0.3) with the following parameters: ppm: 8, peak width: c(5, 24), snthresh: 1, prefilter: c(1, 200), mzCenterFun: “wMeanApex3”, integrate: 1, mzdiff: 0.4, fitgaus: TRUE, scanrange: c(700, 5000) and noise: 0. The XCMS methods 'group' and 'rector' in two iterations were used for peak alignment and retention time correction. The parameters were as follows: method: “nearest”, mzVsRTbalance: 15, mzCheck: 0.2, rtCheck: 20, kNN: 15 for the first iteration and kNN: 10 for the second grouping. Further parameters were missing: 3, extra: 2, smooth: “loess”, span: 0.2, family: “symmetric”, plottype: “mdevden” and missing: 1, extra: 1 for the second iteration of rector. A final grouping step was carried out using the same group parameters as for the second iteration. Finally, missing values were calculated using the 'fillpeaks' method.

Retention times of available CoA standards and the accurate masses of M + 2H ions were used for peak identification. If no synthetic standard was available for a certain compound, both calculation of molecular mass from M + 2H and M + H ions and sum formula prediction from the accurate mass and isotopic pattern were applied for identification using the DataAnalysis software. Whenever possible, MS^2^ fragmentation was used to confirm the presence of the CoA moiety.

### Determination of mass isotopomer distribution

GC/MS data were converted into the CDF-format. For identification of labeled peaks and determination of the corresponding MIDs, chromatograms with the labeled and the unlabeled data were loaded into the software *Non-targeted Tracer Fate Detection 1*.*0* [19]. The deconvolution was performed with a peak threshold of 15, a minimal peak height of 5 and a deconvolution width of 8. For isotope detection the settings were as follows: required amount of label (%): 5, sensitivity: 0, minimal R2: 0.95, max fragment deviation: 0.20, required number of labeled fragments: 1, M1 Correction: 1.0934.

For better peak identification and additional MID determination, the data were processed with the software MetaboliteDetector 2.5 [31] in parallel as described before [24]. MIDs were determined by using the MID wizard and a user-defined spectra library with sum formulas for given fragments.

The MIDs for HPLC/MS data were determined manually as followed: for every identified peak, the M+2H ion and its isomere data were extracted and averaged over the technical replicates. The data were scaled to the most abundant mass and corrected for the natural occurrence of ^13^C according to the number of carbon atoms in the metabolite. Afterward, all labeling below 5% was removed and the data were scaled to the sum of the isotopic peaks. For a better interpretation, MIDs of CoA derivatives were additionally corrected for the determined labeling of the coenzyme A according to the M+1 and M+2 correction for the natural ^13^C occurrence.

## Results & discussion

First, the results for the anterior L-lysine degradation to glutaryl-CoA are described and discussed. In the second part, the results for the posterior L-lysine degradation are presented showing for the first time that the ethylmalonyl-CoA-pathway is involved in this process and discussing influxes and effluxes of the TCA cycle.

### Anterior L-lysine degradation to glutaryl-CoA

**Fig 1:**
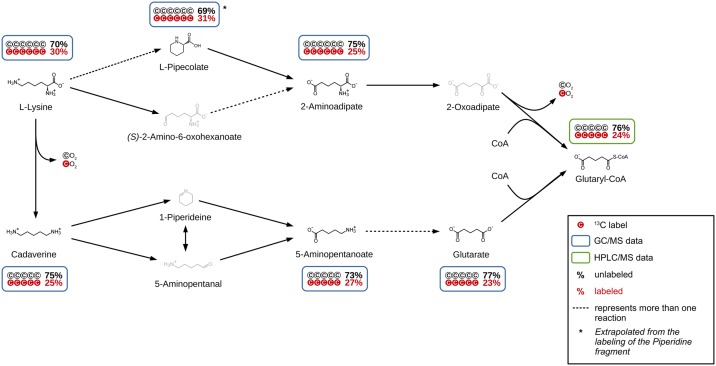
Anterior lysine degradation pathway until glutaryl-CoA. The labeling of all measurable intermediates shows that both pathways are active in *P*. *inhibens* during catabolization of L-lysine as the sole carbon source. Additionally, L-pipecolate was identified to be part of the L-lysine degradation in *P*. *inhibens* for the first time.

For a detailed investigation of lysine degradation in *P*. *inhibens* DSM 17395, a spiking experiment with cells adapted to L-lysine as the sole carbon source was carried out. The cultures were spiked with a ratio of approximately 25% uniformly ^13^C labeled to 75% unlabeled lysine and incubated for 15 minutes. In parallel, cultures were treated with 100% unlabeled lysine and prepared as reference. By using the non-targeted tracer fate detection software (NTFD, [19]), 85 labeled compounds could be detected. While 54 of these compounds could be identified, 31 compounds remained unidentified (S2 Table). The MID of all detectable and identified metabolites of the two anterior lysine degradation pathways was determined (Fig 1). The relative amount of fully ^13^C-labeled compounds ranges between 23% and 31%.

Cadaverine (25% 13^13^C_5_), 5-aminopentanoate (27% 13^13^C_5_) and glutarate (23% 13^13^C_5_) were previously described as part of the lysine degradation pathway in *P*. *aeruginosa* [14]. In addition to those also L-pipecolate (31% 13^13^C_5_) and 2-aminoadipate (25% 13^13^C_6_) show a comparable high amount of ^13^C. They are known to be a part of lysine degradation in *P*. *putida* [32]. For L-pipecolate, only an M+5 labeled fragment could be identified as the piperidine ring, as the COOH-group is lost during EI-ionization. Still, it can be assumed that all six carbons of L-pipecolate are labeled accordingly. This led us to the conclusion that this pathway is also used. Other potential degradation pathway intermediates like (*S*)-2-amino-6-oxohexanoate, 2-oxoadipate, 1-piperideine or 5-aminopentanal could not be detected with this method, as the method is limited regarding the stability and pool size (limit of detection) of metabolites.

The high amount of ^13^C_5_ and ^13^C_6_ molecules (23%–31%), similar to the detected labeling for lysine (30% C_6_), led us to the conclusion, that these metabolites are directly derived from lysine. While the degradation of L-lysine via cadaverine was described before [10], degradation via L-pipecolate in parallel has not been observed before in *P*. *inhibens*. Whether the degradation pathway via 2-aminoadipate involves (*S*)-2-amino-6-oxohexanoate, as described by Drueppel et al.[10] remains unclear, since this metabolite could not be detected.

**Fig 2:**
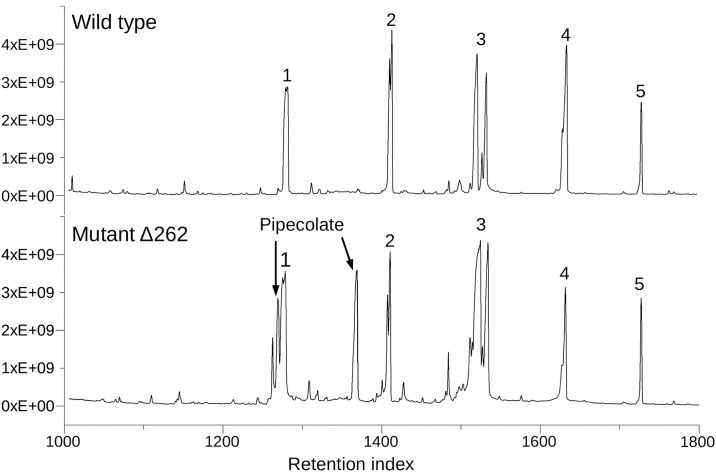
Comparison of the total ion chromatogram of *P*. *inhibens* wild type and Δ262kb curing mutant. 1 phosphate, 2 glutarate, 3 glutamate, 4 5-aminopentanoate, 5 ribitol (internal standard).

The gene coding for the possible pipecolate oxidase (PGA1_262p02210) is localized on the 262 kb plasmid [33]. In order to verify the activity of the pipecolate pathway, the *P*. *inhibens* wild type and a Δ262kb curing mutant were cultivated on L-lysine as the sole carbon source followed by a metabolic comparison of both strains via GC/MS. An accumulation of L-pipecolate would be expected in a mutant missing this plasmid. The resulting total ion chromatograms (TIC) of the wild type and the Δ262kb curing mutant are shown in Fig 2. Two additional large peaks appeared in the TIC of the mutant which were identified as 1TMS- and 2TMS-derivatives of L-pipecolate. This accumulation underlines the assumption that the gene PGA1_262p02210 is coding for a pipecolate oxidase and that the *P*. *inhibens* wild type is able to degrade L-lysine via pipecolate. It could be shown that in *P*. *inhibens* at least two anterior lysine degradation pathways are active during growth on L-lysine as the sole carbon source and that L-pipecolate is a part of it.

A search for the other intermediates of the 11 possible pathways in the chromatograms showed no hints of their presence. Still, in our non-targeted ^13^C analysis we found six unidentified compounds with an M+5 and M+6 labeling over 20%, that could not be identified, due to the low abundance of these compounds (S2 Table “GCMS 15 min”). The high labeling indicates that these compounds are derived from lysine or from metabolites close to the first steps of lysine degradation. However, without knowledge about the identity of these compounds, it is impossible to decide if these are physiological relevant metabolites or by-products that are produced during the process of harvesting and analysis.

### Posterior lysine degradation starting with glutaryl-CoA

For a detailed analysis of the posterior lysine degradation pathway, starting with glutaryl-CoA, a second spiking experiment with *P*. *inhibens* cells growing on L-lysine as the sole carbon source was performed. To achieve a higher labeling in the more distant metabolites, a higher percentage of approximately 40% uniformly ^13^C labeled lysine and an extended incubation time of 60 minutes was applied. The experiment resulted in an unexpected complexity of labeling patterns showing that here again different pathways are used.

Since coenzyme A itself showed a ^13^C-labeling, the MIDs of CoA-derivatives participating in lysine degradation represent a mixture of the ^13^C labeling resulting from the anterior lysine degradation and the labeling of the CoA. For a better interpretation, CoA-derivatives were corrected for the MID of the CoA (S2 Table, HPLC/MS 15min/1h).

**Fig 3:**
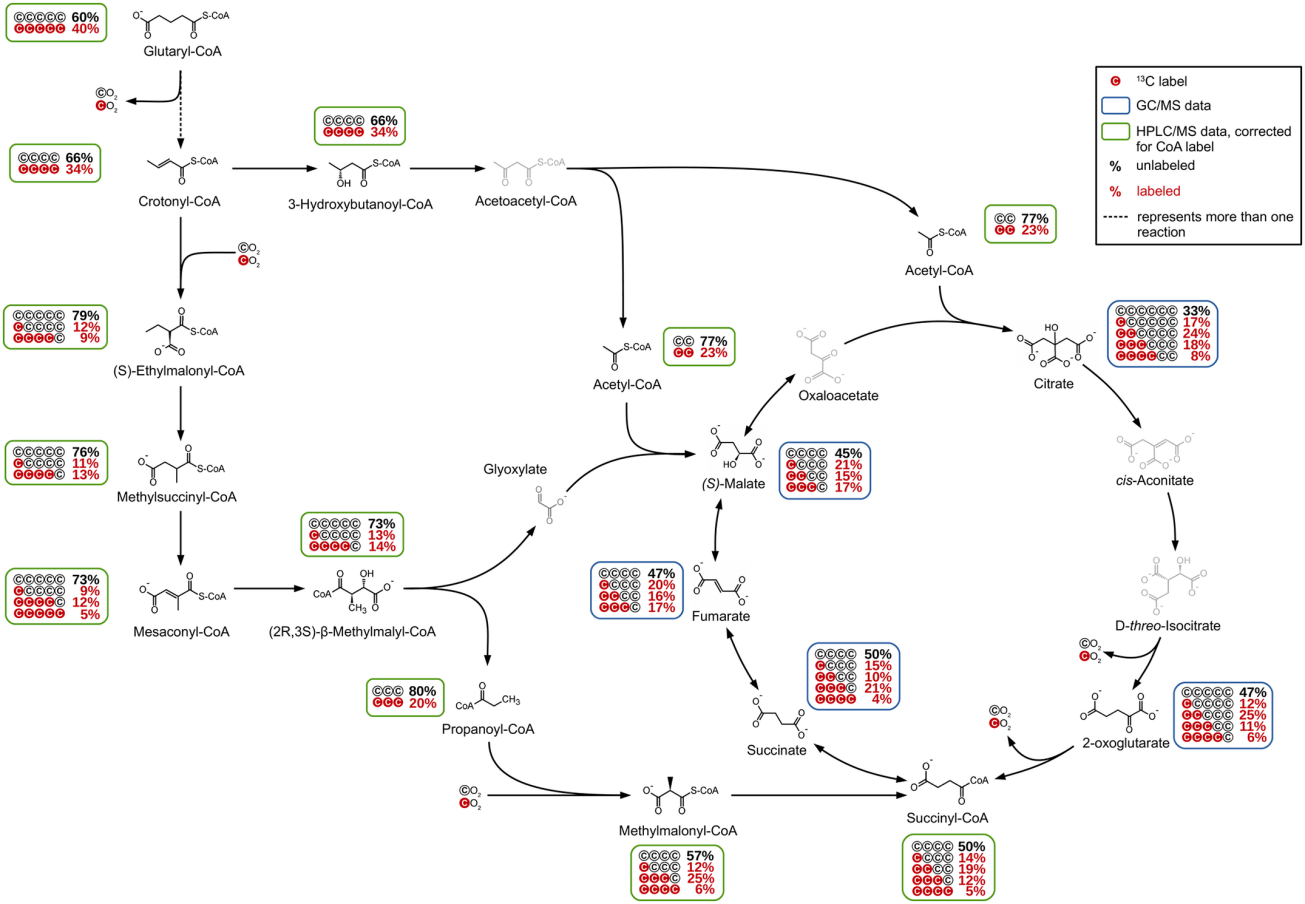
Posterior lysine degradation pathway starting from glutaryl-CoA. The labeling shows that the ethylmalonyl-CoA pathway is active in *P*. *inhibens* during catabolization of L-lysine as the sole carbon source and that three influxes into the TCA cycle can be observed.

The labeling pattern of the detected CoA-derivatives showed that two pathways are used for further degradation of glutaryl-CoA (Fig 3), which showed itself a labeling with an M+0 of 60% and an M+5 of 40%. The posterior lysine degradation starts with a decarboxylation of glutaryl-CoA. The crotonyl-CoA shows an increased amount of unlabeled molecules (M+0) of 66% and a reduced M+4 label of 34%, which might indicate a dilution with unlabeled crotonyl-CoA from an unknown source. Following the known pathway, 3-hydroxybutanoyl-CoA is formed which shows an identical labeling as crotonyl-CoA. During the next reactions, the carbon backbone is split into two molecules of acetyl-CoA with an increased amount of M+0 of 77% and an M+2 of 23%.

This increased amount of unlabeled acetyl-CoA with 77% compared to 66% of 3-hydroxybutanoyl-CoA is unexpected on the first view. It indicates a dilution of the labeled acetyl-CoA pool with unlabeled molecules from a so far not identified source. Based on the assumption that the additional acetyl-CoA was completely unlabeled, an approximate ratio of 3 to 1 of the flux from lysine compared to the other source could be calculated.

Our data indicate that in *P*. *inhibens* the ethylmalonyl-CoA pathway (EMC pathway) participates in the lysine metabolism in addition to the expected one leading via 3-hydroxybutanoyl-CoA and acetyl-CoA into the TCA cycle and further via respiration to carbon dioxide (Fig 3).

In the originally published form, the EMC pathway starts with the condensation of two acetyl-CoA molecules forming crotonyl-CoA and ends with the TCA cycle intermediates (*S*)-malate and succinyl-CoA [34]. For the first steps leading to crotonyl-CoA, this is the opposite direction compared to the glutaryl-CoA degradation described here. This can be excluded due to the absence of M+2 labeling e.g. in hydroxybutanoyl-CoA and crotonyl-CoA. In contrast, crotonyl-CoA resulting from the lysine degradation is directly used as starting point for the EMC pathway in *P*. *inhibens*. This is also supported by the labeling found in the following EMC pathway intermediates, which all showed an M+4 labeling resulting from glutaryl-CoA degradation and M+1 labeling introduced by the carboxylation of unlabeled crotonyl-CoA with labeled carbon dioxide.

Following a number of reactions, the substrates and products of which were all detected in our analysis, the EMC proceeds via the cleavage of (2*R*,3*S*)-β-methylmalyl-CoA into glyoxylate and propanoyl-CoA. Propanoyl-CoA showed a labeling pattern with M+3 of 20% and no detectable M+1 labeling. This is explained by the atom transitions occurring in the EMC pathway, which show that the carbon backbone of propanoyl-CoA originates solely from crotonyl-CoA. In the following reaction, propanoyl-CoA is carboxylated to methylmalonyl-CoA, which again introduces a labeled carbon from carbon dioxide resulting in an additional M+1 and M+4 labeling.

As the retention time of glyoxylate is too short for the used GC/MS method only a theoretical labeling can be calculated. On the basis of atom transitions in the EMC pathway, glyoxylate can consist of (I) fully unlabeled carbon atoms, (II) one labeled carbon from carbon dioxide and one unlabeled carbon from crotonyl-CoA, (III) one unlabeled carbon from carbon dioxide and one labeled carbon from crotonyl-CoA, and (IV) one labeled carbon from carbon dioxide and one labeled carbon from crotonyl-CoA. The latter can be disregarded since the amount of M+5 labeling in (2*R*,3*S*)-β-methylmalyl-CoA is under the defined threshold of 5% labeling (although the M+5 labeling of the mesaconyl-CoA is with 5.2% just above the threshold). In consequence, the expected labeling of glyoxylate resulting from the cleaved (2*R*,3*S*)-β-methylmalyl-CoA would show an M+0 of 73% and an M+1 of 27%. The M+1 of 27% would consist of 13% M+1 originating from carbon dioxide and 14% M+1 originating from crotonyl-CoA. Glyoxylate, as well as methylmalonyl-CoA, were identified as feeding compounds for the TCA cycle (vide infra).

Although the fate of labeled carbon in the EMC pathway could almost be completely followed in our analyses and the atom transitions are well known, the amount of labeled carbon dioxide cannot be determined. Since the medium provides a source of unlabeled carbon dioxide, the available carbon dioxide for the organism is a mixture of molecules that originate from its own metabolism and molecules from the medium. While the amount of molecules originating from the medium will be relatively stable, the amount of labeled carbon dioxide produced during lysine metabolism increases over time (of the labeling experiment). In addition, the occurrence of isotopic effects cannot be generally excluded. Therefore, the calculation of the amount of labeling in the (re-) incorporated carbon dioxide in the cell is impossible.

### TCA cycle influxes

The labeling pattern detected for the TCA cycle intermediates showed that carbon is introduced into the cycle not solely via acetyl-CoA and citrate but also via the EMC pathway. As expected, citrate showed a high amount of M+2 labeling, resulting from acetyl-CoA (Table 1). However, the measured M+1 and M+3 labeling of citrate showed noticeable higher values than it would be expected with acetyl-CoA as the sole carbon influx. Decarboxylation of labeled carbon atoms of citrate by isocitrate dehydrogenase leads to a decrease of labeling in 2-oxoglutarate, which resulted in a strongly increased M+0 labeling compared to citrate. Since the two carbon atoms introduced via acetyl-CoA are not lost via decarboxylation in the first round of the TCA cycle, no decrease of the M+2 label in 2-oxoglutarate is observable.

**Table 1:** Mass isotopomer distribution of the TCA cycle intermediates.

Metabolite	M+0 (SD)	M+1 (SD)	M+2 (SD)	M+3 (SD)	M+4 (SD)
Citrate	33% ±1.53	17% ±1.56	24% ±1.45	18% ±1.1	8% ±1.13
2-Oxoglutarate	47% ±0.15	12% ±0.34	25% ±1.23	11% ±0.22	6% ±1.99
Succinyl-CoA	50% ±2.08	14% ±1.68	19% ±3.08	12% ±2.73	5% ±1.69
Succinate	50% ±0.09	15% ±0.14	10% ±1.21	21% ±0.22	4% ±1.09
Fumarate	47% ±0.15	20% ±0.48	16% ±0.18	17% ±0.74	
Malate	45% ±0.05	21% ±0.43	15% ±0.02	17% ±0.33	

For GC/MS data n = 3, for HPLC/MS data (Succinyl-CoA) n = 4. Not determined mass isotopologue fractions are not displayed. SD = relative standard deviation.

During the next decarboxylation step to succinyl-CoA, the M+0 share remained almost unchanged and only a slight decrease of labeled molecules was observed. As can be seen in Table 1, the determined labeling data for succinyl-CoA showed an increased error compared to the other TCA cycle intermediates. A comparison of the labeling of succinyl-CoA and succinate showed identical amounts of M+0, M+1, and M+4 for both metabolites, whereas M+2 and M+3 showed an almost reversed ratio between succinyl-CoA and succinate. Since the reaction of succinyl-CoA to succinate involves no changes in the carbon backbone, this observation was unexpected. Because of the smaller experimental error, the labeling data of succinate are more reliable and were thus assumed to reflect the true labeling expected for succinyl-CoA. This was further supported by the increase of M+3 labeling in succinate compared to 2-oxoglutarate, which can be explained by an influx from methylmalonyl-CoA showing an amount of M+3 of 25%.

Although the following reactions via fumarate to malate comprise no changes in the carbon backbone, the MIDs of fumarate and malate showed increased amounts of M+1 and M+2 (Table 1) from 15% to 20% and 10% to 16% compared to succinate, while the determined amount of M+3 decreased from 21% to 17%. This observation can be explained by a carbon influx via glyoxylate from the EMC pathway at the point of malate. As described before, based on the atom transitions in the EMC pathway, the theoretical labeling for glyoxylate was calculated. Accordingly, the labeling of malate resulting from the reaction of glyoxylate with acetyl-CoA would comprise an M+0 of 56%, an M+1 of 21%, an M+2 of 17% and an M+3 of 6%. Compared to the determined labeling for malate, the calculated values show almost identical amounts of M+1 and M+2, whereas for M+0 a higher amount and for M+3 a slightly lower amount was calculated. While the difference in the M+0 can be explained by the summation of deviations in the M+0 of glyoxylate and acetyl-CoA, the differences in M+3 could originate from the flux from succinate introducing an M+3 amount of 21% into the pool of fumarate and malate. Altogether, it is assumed that the labeling determined for malate results from a combination of fluxes from succinate (M+3) and glyoxylate + acetyl-CoA (M+1 and M+2).

Based on the assumption that oxaloacetate and malate comprise identical labeling (oxaloacetate not detected by GC/MS), a hypothetical citrate labeling was calculated with the experimentally determined labeling of malate and acetyl-CoA. The hypothetical citrate labeling would be as follows: M+0 of 35%, M+1 of 15%, M+2 of 22%, M+3 of 18%, M+4 of 4% and M+5 of 4%. The calculated values of citrate labeling were almost identical to the determined ones, except for M+4 and M+5, which show higher errors due to their low abundances. This finding strongly underlines the accuracy of the experimentally determined data.

Due to the complexity of the TCA cycle interactions with the whole metabolism, the interpretation of the labeling data is challenging and in some cases even impossible. Since the labeling of carbon dioxide in the cell is unknown only the general tendency of the downgrade of labeling leading to an increase of the amount of M+0 can be described. Nonetheless, the influence of the three influxes at citrate, succinyl-CoA, and malate are clearly observable. Although the determined labeling of succinyl-CoA cannot be fully explained, the high M+3 labeling in succinate, fumarate, and malate strongly indicate the introduction of M+3 labeled carbon via methylmalonyl-CoA. Furthermore, the increase of M+1 in malate compared to succinyl-CoA and succinate points out an influx of M+1 via glyoxylate into the TCA cycle.

### TCA cycle effluxes

**Fig 4:**
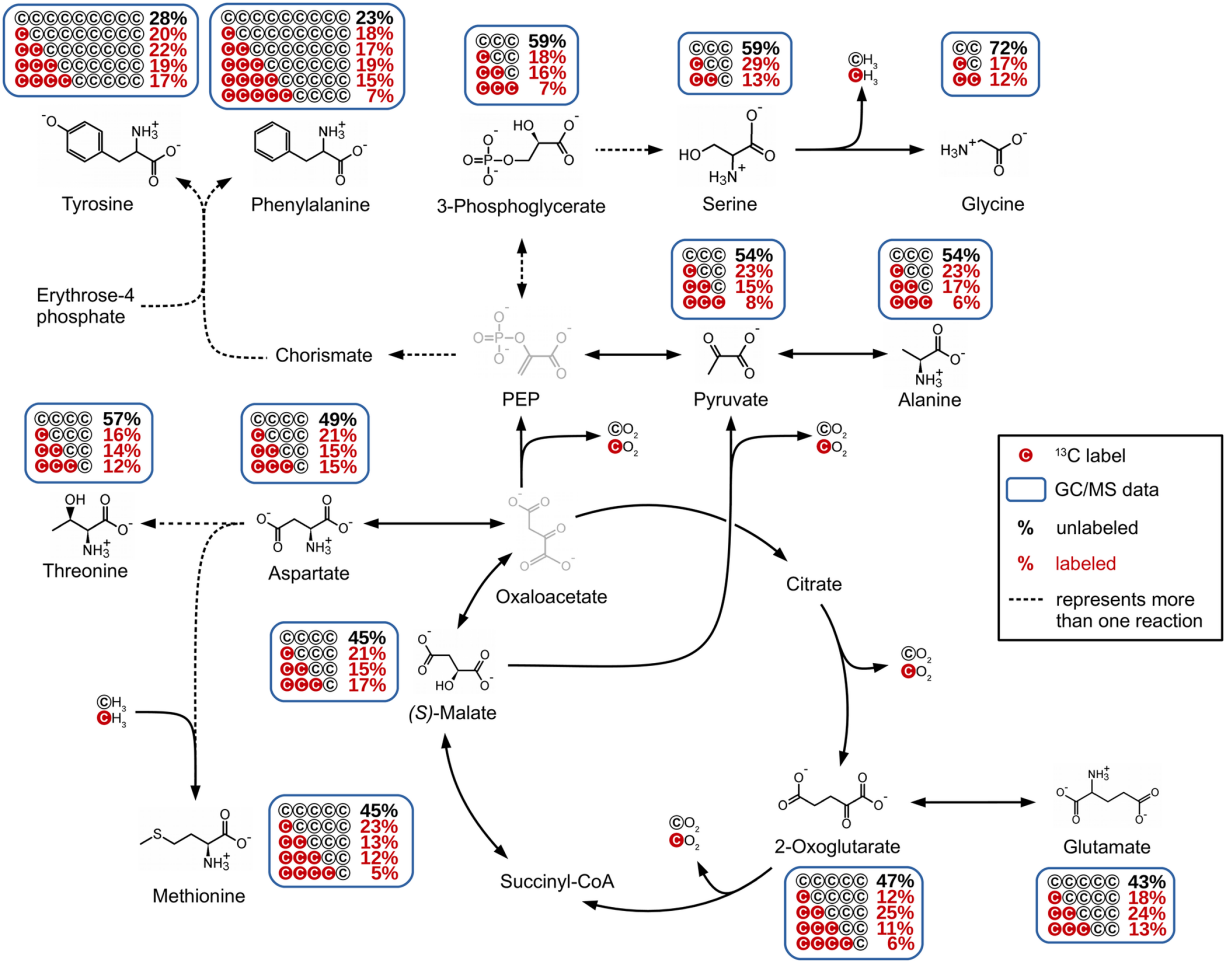
Overview of the observed TCA cycle effluxes.

Not only three influx, but also three efflux points were observed for the TCA cycle in the labeling experiments (Fig 4). Since the reaction from 2-oxoglutarate to glutamate involves no changes in the carbon backbone, the labeling of 2-oxoglutarate and glutamate showed a similar labeling pattern. The same holds true for the labeling of malate and aspartate. Aspartate is the starting point for a number of pathways, including amino acid syntheses. We were able to determine the labeling of threonine and methionine. The increase of the amount of unlabeled molecules from 49% in aspartate to 57% in threonine indicates that these metabolites are not closely connected. A possible reason would be that the threonine pool includes molecules from another source e.g. glycine. Another explanation would be that these two metabolites are not in equilibrium, possibly caused by the stop of the metabolism during harvesting and processing. In contrast to this observation, the labeling of methionine showed a slight decrease of the M+0 to 45%, which results from the addition of one methyl group by methionine synthase. This was also reflected by an increased M+4 labeling of 5%, which was below the 5% threshold in the case of aspartate.

Contrary to glutamate and aspartate, the efflux from the TCA cycle to pyruvate either via decarboxylation of oxaloacetate to phosphoenolpyruvate or directly via decarboxylation of malate causes changes in the labeling pattern. Decarboxylation of labeled carbon atoms causes a decrease in labeling pattern, resulting in an increased M+0 (54%) and a decreased M+3 (8%) amount for pyruvate compared to malate.

While alanine showed an identical labeling to pyruvate, 3-phosphoglycerate showed a slightly increased amount of M+0 and decreased amount of M+1. Since phosphoenolpyruvate could not be detected, it remains unclear whether this alteration originates from the decarboxylation of oxaloacetate or from another source (as for example recycled glycogen). A comparison of the labeling of serine with 3-phosphoglycerate showed an identical M+0 of 59%, while pyruvate showed an M+0 of 54%. This indicates a closer connection of serine to 3-phosphoglycerate than to pyruvate. Compared to 3-phosphoglycerate (18%), serine showed a higher M+1 of 29%, which could result from another source that combines two labeled molecules and therefore raises the labeling of M+1. Due to the multiple possible origins for serine this cannot be decided. The same holds true for glycine. The reaction from serine to glycine involves a loss of one carbon atom, which results in an increase of unlabeled molecules as described for the decarboxylation steps before. In *P*. *inhibens*, glycine can also be produced by other reactions like the degradation of threonine, or can be formed from glyoxylate. However, based on the labeling patterns, it is not possible to differentiate these possibilities and it is probable that the labeling of glycine reflects a mixed carbon labeling originating from different precursors.

Among all identified metabolites, tyrosine and phenylalanine showed the lowest determined M+0 labeling. Since the biosynthesis of these amino acids incorporates two molecules of phosphoenolpyruvate and one molecule of erythrose-4-phosphate, the amount of unlabeled molecules is significantly reduced. Accordingly, the resulting labeling is complex and since neither the labeling of phosphoenolpyruvate nor the labeling of erythrose-4-phosphate are known, no calculations can be done. The small differences in the labeling of tyrosine and phenylalanine can be attributed to the missing M+5 labeling of tyrosine which could not be determined due to its low abundance in the GC/MS chromatograms.

In general, the differences in the abundance of metabolites and therefore the limitation of sensitivity for certain mass fragments containing the complete carbon backbone are a minor drawback of the described method. As can be seen for tyrosine, the missing M+5 is changing the complete labeling. Assuming an M+5 of 7%, according to phenylalanine, the labeling would change as follows: M+0 from 28% to 25%, M+1 from 20% to 17%, M+2 from 22% to 19%, M+3 from 19% to 17%, M+4 from 17% to 15% and M+5 from 0% to 7%. In consequence, the amount of M+0 is often overestimated in metabolites, for which the MID is incomplete or the labeling of isotopologues lies below the 5% threshold of the method.

## Conclusion

We present a way to analyze metabolic pathways allowing a very detailed insight into complex metabolic pathways without a priori knowledge. The application of the labeling-based pathway analysis method shows that lysine catabolism in *Phaeobacter inhibens* DSM 17395 is much more complicated than anticipated applying other methods.

Using the non-targeted tracer fate detection software [19] for the analysis of GC/MS data not only allows the detection of new pathways but also supports the identification of yet unknown metabolites and their assignment to specific pathways.

By the combination of GC/MS and HPLC/MS analyses, we were able to follow in detail the fate of labeled carbon from L-lysine in more than 15 metabolites before entering the TCA cycle, giving an almost gapless picture of L-lysine degradation in *P*. *inhibens*. This reveals a complex degradation pathway pattern involving two parallel ways for the degradation of L-lysine via cadaverine and L-pipecolate. Although very uncommon, this is not unique amongst bacteria. A comparable complex L-lysine metabolism can be found for *P*. *putida* [15] involving 5-aminopentanoate and L-pipecolate in two parallel pathways. This could mean that the existence of several lysine degradation pathways could be more common within bacteria than so far noticed, due to the usually targeted and hypothesis-driven approaches applied.

Since the experiments described in this manuscript were designed to analyze the fate of lysine and not to analyze fluxes, the data show limitations which did not allow for calculation of corresponding fluxes at branching points. Therefore we are not able to make predictions regarding the usage of the uncovered pathways. For the anterior lysine pathway, the growth of the Δ262kb curing mutant with a disrupted pipecolate-pathway might indicate a minor role of this pathway but is also related to the absence of the growth inhibiting antimicrobial compound tropodithietic acid (TDA), whose biosynthesis is encoded on the 262kb plasmid as well [33]. For the EMC pathway, the labeling pattern of the TCA cycle intermediates strongly indicate, that this pathway is active during L-lysine metabolization and that the EMC pathway is used to introduce carbon into the TCA cycle at the points of succinyl-CoA and malate.

Nevertheless, the labeling data provided a comprehensive picture of the lysine metabolism in *P*. *inhibens*. The sometimes unexpected labeling of intermediates described in this work would not have been found in a targeted approach and indicates the drawback of classical metabolic flux analyses relying often on labeling information of only a few or single metabolites for calculations of complex pathways. In contrast, the high accuracy of the experimentally determined data presented here could be impressively shown in the high accordance of calculated and determined labeling for citrate.

## Supporting information

S1 Table Overview growth data. (XLSX)

S2 Table Overview ^13^C labeling GC/MS and HPLC/MS data. (XLS)

## References

[1] Brinkhoff T, Giebel HA, Simon M. Diversity, ecology, and genomics of the *Roseobacter* clade: A short overview. Arch Microbiol. 2008;189(6):531–9. 10.1007/s00203-008-0353-y 18253713

[2] Biebl H, Allgaier M, Tindall BJ, Koblizek M, Lünsdorf H, Pukall R, et al *Dinoroseobacter shibae* gen. nov., sp. nov., a new aerobic phototrophic bacterium isolated from dinoflagellates. Int J Syst Evol Microbiol. 2005;55(3):1089–96. 15879238 10.1099/ijs.0.63511-0

[3] Martens T, Heidorn T, Pukal R, Simon M, Tindall BJ, Brinkhoff T. Reclassification of *Roseobacter gallaeciensis* Ruiz-Ponte et al. 1998 as *Phaeobacter gallaeciensis* gen. nov., comb. nov., description of *Phaeobacter inhibens* sp. nov., reclassification of *Ruegeria algicola* (Lafay et al. 1995) Uchino et al. 1999 as *Marinovu*. Int J Syst Evol Microbiol. 2006;56(6):1293–304. 16738106 10.1099/ijs.0.63724-0

[4] Geng H, Belas R. Molecular mechanisms underlying *roseobacter*-phytoplankton symbioses. Curr Opin Biotechnol [Internet]. 2010;21(3):332–8. Available from: 10.1016/j.copbio.2010.03.013 20399092

[5] Seyedsayamdost MR, Case RJ, Kolter R, Clardy J. The Jekyll-and-Hyde chemistry of *Phaeobacter gallaeciensis*. Nat Chem. 2011;3(4):331–5. 10.1038/nchem.1002 21430694 PMC3376411

[6] Zech H, Hensler M, Koßmehl S, Drüppel K, Wöhlbrand L, Trautwein K, et al Adaptation of *Phaeobacter inhibens* DSM 17395 to growth with complex nutrients. Proteomics. 2013;13(18–19):2851–68. 10.1002/pmic.201200513 23613352

[7] Wiegmann K, Hensler M, Wöhlbrand L, Ulbrich M, Schomburg D, Rabus R. Carbohydrate catabolism in *Phaeobacter inhibens* DSM 17395, a member of the marine *Roseobacter* clade. Appl Environ Microbiol. 2014;80(15):4725–37. 10.1128/AEM.00719-14 24858085 PMC4148808

[8] Zech H, Hensler M, Koßmehl S, Drüppel K, Wöhlbrand L, Trautwein K, et al Dynamics of amino acid utilization in *Phaeobacter inhibens* DSM 17395. Proteomics [Internet]. 2013 10 [cited 2014 Feb 13];13(18–19):2869–85. Available from: http://www.ncbi.nlm.nih.gov/pubmed/23625753 23625753 10.1002/pmic.201200560

[9] Gram L, Rasmussen BB, Wemheuer B, Bernbom N, Ng YY, Porsby CH, et al *Phaeobacter inhibens* from the *Roseobacter* clade has an environmental niche as a surface colonizer in harbors. Syst Appl Microbiol [Internet]. 2015;38(7):483–93. Available from: 10.1016/j.syapm.2015.07.006 26343311

[10] Drueppel K, Hensler M, Trautwein K, Koßmehl S, Wöhlbrand L, Schmidt-Hohagen K, et al Pathways and substrate-specific regulation of amino acid degradation in *Phaeobacter inhibens* DSM 17395 (archetype of the marine *Roseobacter* clade). Environ Microbiol [Internet]. 2014 1 [cited 2014 Feb 21];16(1):218–38. Available from: http://www.ncbi.nlm.nih.gov/pubmed/24165547 24165547 10.1111/1462-2920.12276

[11] Caspi R, Billington R, Ferrer L, Foerster H, Fulcher CA, Keseler IM, et al The MetaCyc database of metabolic pathways and enzymes and the BioCyc collection of pathway/genome databases. Nucleic Acids Res. 2016;44(D1):D471–80. 10.1093/nar/gkv1164 26527732 PMC4702838

[12] Higashino K, Tsukada K, Lieberman I. Saccharopine, a product of lysine breakdown by mammalian liver. Biochem Biophys Res Commun. 1965;20(3):285–90. 5856802 10.1016/0006-291x(65)90361-x

[13] Foster JW. *Escherichia coli* acid resistance: tales of an amateur acidophile. Nat Rev Microbiol. 2004;2(11):898–907. 10.1038/nrmicro1021 15494746

[14] Fothergill JC, Guest JR. Catabolism of L-Lysine by *Pseudomonas aeruginosa*. Culture. 1977;99:139–55. 10.1099/00221287-99-1-139 405455

[15] Revelles O, Espinosa-Urgel M, Fuhrer T, Sauer U, Ramos JL. Multiple and interconnected pathways for l-lysine catabolism in *Pseudomonas putida* KT2440. J Bacteriol. 2005;187(21):7500–10. 10.1128/JB.187.21.7500-7510.2005 16237033 PMC1272968

[16] Calvin M, Benson AA. The Path of Carbon in Photosynthesis. Science. 1948;107:476–80. 10.1126/science.107.2784.476 17760010

[17] Entner N, Doudoroff M. Glucose and gluconic acid oxidation of *Pseudomonas saccharophila*. J Biol Chem. 1952;196:853–62. 12981024

[18] Kind S, Becker J, Wittmann C. Increased lysine production by flux coupling of the tricarboxylic acid cycle and the lysine biosynthetic pathway-Metabolic engineering of the availability of succinyl-CoA in *Corynebacterium glutamicum*. Metab Eng [Internet]. 2013;15(1):184–95. Available from: 10.1016/j.ymben.2012.07.005 22871505

[19] Hiller K, Wegner A, Weindl D, Cordes T, Metallo CM, Kelleher JK, et al NTFD—A stand-alone application for the non-targeted detection of stable isotope labeled compounds in GC/MS data. Bioinformatics [Internet]. 2013 3 11;11–3. Available from: http://www.ncbi.nlm.nih.gov/pubmed/23479350 10.1093/bioinformatics/btt119 PMC3634188 23479350

[20] Chokkathukalam A, Jankevics A, Creek DJ, Achcar F, Barrett MP, Breitling R. MzMatch-ISO: An R tool for the annotation and relative quantification of isotope-labelled mass spectrometry data. Bioinformatics. 2013;29(2):281–3. 10.1093/bioinformatics/bts674 23162054 PMC3546800

[21] Huang X, Chen YJ, Cho K, Nikolskiy I, Crawford PA, Patti GJ. X13CMS: Global tracking of isotopic labels in untargeted metabolomics. Anal Chem. 2014;86(3):1632–9. 10.1021/ac403384n 24397582 PMC3982964

[22] Trautwein K, Will SE, Hulsch R, Maschmann U, Wiegmann K, Hensler M, et al Native plasmids restrict growth of *Phaeobacter inhibens* DSM 17395: Energetic costs of plasmids assessed by quantitative physiological analyses. Environ Microbiol. 2016;18(12):4817–29. 10.1111/1462-2920.13381 27233797

[23] Zech H, Thole S, Schreiber K, Kalhöfer D, Voget S, Brinkhoff T, et al Growth phase-dependent global protein and metabolite profiles of *Phaeobacter gallaeciensis* strain DSM 17395, a member of the marine *Roseobacter*-clade. Proteomics [Internet]. 2009 7 [cited 2013 Jan 30];9(14):3677–97. Available from: http://www.ncbi.nlm.nih.gov/pubmed/19639587 19639587 10.1002/pmic.200900120

[24] Abu Dawud R, Schreiber K, Schomburg D, Adjaye J. Human embryonic stem cells and embryonal carcinoma cells have overlapping and distinct metabolic signatures. PLoS One. 2012;7(6):1–11. 10.1371/journal.pone.0039896 PMC3387229 22768158

[25] Reimer LC, Spura J, Schmidt-Hohagen K, Schomburg D. High-Throughput Screening of a *Corynebacterium glutamicum* Mutant Library on Genomic and Metabolic Level. PLoS One [Internet]. 2014 1 [cited 2014 Feb 13];9(2):e86799 Available from: http://www.pubmedcentral.nih.gov/articlerender.fcgi?artid=3913579&tool=pmcentrez&rendertype=abstract 24504095 10.1371/journal.pone.0086799 PMC3913579

[26] Peyraud R, Kiefer P, Christen P, Massou S, Portais J-C, Vorholt JA. Demonstration of the ethylmalonyl-CoA pathway by using 13C metabolomics. Proc Natl Acad Sci USA [Internet]. 2009;106(12):4846–51. Available from: http://www.ncbi.nlm.nih.gov/pubmed/19261854 19261854 10.1073/pnas.0810932106 PMC2660752

[27] Wolf J, Stark H, Fafenrot K, Albersmeier A, Pham TK, Müller KB, et al A systems biology approach reveals major metabolic changes in the thermoacidophilic archaeon *Sulfolobus solfataricus* in response to the carbon source L-fucose versus D-glucose. Mol Microbiol. 2016; 10.1111/mmi.13498 27611014

[28] Smith CA, Want EJ, O’Maille G, Abagyan R, Siuzdak G. XCMS: processing mass spectrometry data for metabolite profiling using nonlinear peak alignment, matching, and identification. Anal Chem [Internet]. 2006 2 1;78(3):779–87. Available from: http://www.ncbi.nlm.nih.gov/pubmed/16448051 16448051 10.1021/ac051437y

[29] Benton HP, Wong DM, Trauger SA, Siuzdak G. XCMS2: Processing Tandem Mass Spectrometry Data for Metabolite Identification and Structural Characterization. 2008;80(16):6382–9. 10.1021/ac800795f PMC2728033 18627180

[30] Tautenhahn R, Patti GJ, Rinehart D, Siuzdak G. XCMS Online: a web-based platform to process untargeted metabolomic data. Anal Chem [Internet]. 2012 6 5;84(11):5035–9. Available from: http://www.ncbi.nlm.nih.gov/pubmed/22533540 22533540 10.1021/ac300698c PMC3703953

[31] Hiller K, Hangebrauk J, Ja C, Spura J, Schreiber K. MetaboliteDetector: Comprehensive Analysis Tool for Targeted and Nontargeted GC / MS Based Metabolome Analysis. Anal Biochem. 2009;81(9):3429–39. 10.1021/ac802689c 19358599

[32] Revelles O, Wittich RM, Ramos JL. Identification of the initial steps in D-lysine catabolism in *Pseudomonas putida*. J Bacteriol. 2007;189(7):2787–92. 10.1128/JB.01538-06 17259313 PMC1855791

[33] Will SE, Neumann-Schaal M, Heydorn RL, Bartling P, Petersen J, Schomburg D. The limits to growth—energetic burden of the endogenous antibiotic tropodithietic acid in *Phaeobacter inhibens* DSM 17395. PLoS One. 2017;1–11. 10.1371/journal.pone.0177295 PMC5421792 28481933

[34] Erb TJ, Fuchs G, Alber BE. (2S)- Methylsuccinyl-CoA dehydrogenase closes the ethylmalonyl-CoA pathway for acetyl-CoA assimilation. Mol Microbiol. 2009;73(6):992–1008. 10.1111/j.1365-2958.2009.06837.x 19703103

